# Molecular identification of *Candida* species isolated from candiduria and its risk factors in neonates and children

**DOI:** 10.18502/cmm.7.3.7799

**Published:** 2021-09

**Authors:** Fariba Shirvani, Mahsa Fattahi

**Affiliations:** 1 Pediatric Infections Research Center, Research Institute for Children Health, Shahid Beheshti University of Medical Sciences, Tehran, Iran; 2 Center for Research and Training in Skin Diseases and Leprosy, Tehran University of Medical Sciences, Tehran, Iran

**Keywords:** Candiduria, *Candida* species, Children, Neonates, Risk factors

## Abstract

**Background and Purpose:**

The present study was performed to raise attention on the frequency of *Candida* spp. and evaluation of risk factors of candiduria in neonates and children.

**Materials and Methods:**

In total, 60 urine samples were collected from the suspected neonates and children. Identification of *Candida* at species level was performed using the polymerase chain reaction-restriction fragment length polymorphism approach.

**Results:**

The restriction fragment length polymorphism fingerprint analysis revealed that *Candida parapsilosis* (n=17; 28.33 %) is the most prevalent isolated species followed by *Candida albicans* (n=9; 15%), *Candida tropicalis* (n=4; 9.52%), and *C. glabrata* (n=2; 4.76%). All of the *C. albicans* and *C. parapsilosis* complex strains were identified as *C. albicans* with *HWP1* gene primers and using the *NlaIII* restriction enzyme activity, respectively. In this study, none of the mentioned factors was the cause of infection, but they could be considered risk factors. The mean hospital stay was 21 days (range: 7-21 days). More than 90% of the patients had a urinary catheter, and about 26% of them received antibiotics. Regarding the risk factors, there was no significant difference between the two groups of candidiasis in terms of *C. albicans* and non-albicans *Candida* (*P*<0.01).

**Conclusion:**

Candiduria has always been a challenging issue, especially in children admitted to hospitals. Outcome of candiduria in patients with generally healthy is little.

## Introduction

Urinary tract infection (UTI) is known as one of the major nosocomial infections [ 1 ]. In total, 90% of cases are caused by bacteria and 10-15% of them are caused by fungi, particularly *Candida* spp. [ 2 ]. The UTI may involve the upper urinary tract (e.g., pyelonephritis) or the lower urinary tract (e.g., cystitis). It may be challenging, if not unreasonable, to separate pyelonephritis from cystitis based on clinical signs, particularly in newborns and young children. The UTIs as a result of *Candida* spp. have become a challenging matter in intensive care units [ 3 ].

The occurrence of *Candida* spp. in the urine may characterize several disorders that need precise interpretation of the report, ranging from specimen impurity to UTI, including disseminated candidiasis [ 4 ]. Some factors, such as older age, female gender, use of antibiotics, urinary catheters, diabetes, immunosuppressive treatment, and prolonged hospital stay are known as the key risk factors for candiduria [ 5 , 6 ]. As soon as the presence of *Candida* in the urine is approved, a precise medicinal assessment should be completed to detect symptoms indicative of other disorders, such as diabetes mellitus, genitourinary structural abnormalities, decreased renal function, and metabolic syndromes [ 7 ]. 

Differences in *Candida* spp. are associated with disease severity and diversity in antifungal sensibility outlines. Therefore, the acurate identification of *Candida* spp., as well as the determination of sensitiviy patern to the main antifungal agents lead to choose aproplate treatment regime. Applied molecular approaches to discriminate etiological agents at the species level result in the progress of the difficulty of symptoms and limit the diffusion of the disease trough prescription of a suitable antifungal regime.

Candida can be normally diagnosed with microscopic examination and other phenotypic methods, including chlamydoconidia production and chromogenic medium (CHROMagar). Moreover, the absorption and fermentation of carbohydrates help to identify *Candida* spp. [ 8 ]. These methods are very time-consuming and require several days to identify various *Candida* spp. [ 8 ]. Other disadvantages of such tests include their low specificity and dependence on phenotypic properties. Tests based on the phenotypic property are more subject to environmental changes than the tests based on genotyping property.

Therefore, the present study was performed to show the frequency of *Candida* spp. in the urine sample of the subjects who were admitted to a hospital in Tehran, Iran during 2019. The polymerase chain reaction (PCR)- Restriction fragment length polymorphism (RFLP) method was used to evaluate the risk factors.

## Materials and Methods

### Study design 

After eligible subjects received detailed explanations about the study, informed consent was obtained from them.

### Subjects and procedure

This cross-sectional and experimental study was conducted in 2019. The hospitalized children (from Imam Hossain Hospital, Tehran, Iran) suspected of sepsis or candiduria and those with pyuria were enrolled in this study. It should be mentioned that the subjects who had positive cutler of bacteria were excluded. The Ethics Committee of Shahid Beheshti University of Medical Sciences, Tehran, Iran approved this study (Ethics Code: IR.SBMU.MSP.REC.1398.631).

In total, 60 first-void midstream urine samples or specimens were collected by urinary catheter from hospitalized patients who referred to the mycology research center. The demographic characteristics, history, and symptoms of patients were also recorded.

### Initial identification of Candida spp.

Urine samples were centrifuged at 1500 rpm for 10 min and urine direct examination was performed to detect *Candida* fungal elements in urine sediment. Initial identification of *Candida* spp. was performed based on colony color and yeast counts of more than 10^5^ colony-forming unit/ml on CHROM agar *Candida* medium (CHROM agar, France) and sabouraud dextrose agar (Merck, Germany) at 35 °C for 24 h. The ability of pseudohyphae formation in all *Candida* spp. has been confirmed using corn meal agar plus tween 80 culture.

### Molecular identification of *Candida* spp.

Finally, definite identification of *Candida* spp. was confirmed by PCR-RFLP. This identification was based on the amplification of ITS1-5.8SrDNA-ITS2 region from rRNA complex with pan fungal primers ITS1-ITS4 that were synthesized by Bioneer company (Korea). The DNA extraction from *Candida* isolates was performed using the DNA tissue kit (Qiagen, Germany). The PCR was carried out with a PCR reaction mixture, including 1 μl of the extracted DNA, 10 µl of Taq DNA Polymerase Master Mix RED (Ampliqon, Denmark), 1 µl of each ITS1 (5´-TCCGTAGGTGAACCTGCGG-3´) and ITS4 (5´-TCCTCCGCTTATTGATATGC-3´) oligonucleotide primers, and 12 µl of water.

The PCR cycling parameters were 94 °C for 5 min, 35 cycles of denaturation for 1 min at 95 °C, annealing for 1 min at 56 °C, extension for 90 sec at 72 °C, and a final extension for 7 min at 72 °C. The PCR products were visualized by 1.5% agarose gel electrophoresis in Tris-borate-EDTA buffer (Merck, Germany) and stained.

All digestion reactions were performed in 15 μl of a mixture containing 2 μl of 10× buffer, 2 μl of the enzyme, 10 μl of topoisomerase amplicon, and enough ultrapure water (1 μl) to reach the final volume. Restriction enzyme digestions were performed with *MSP* I at 37 °C for 8-10 h [ 9 ]. The PCR amplicons and restriction enzyme digestion products were loaded in 2% (w⁄v) agarose gel in the presence of red gel (0.5 µg mL^−1^). After running for 1.5 h at 90 V cm^−1^. A DNA molecular weight marker and 100 bp ladder (Fermentas, USA) were used. 

The *C. albicans* complex and *C. parapsilosis* complex were differentiated using the *HWP1* gene amplification [ 10 ] and PCR-RFLP with *NlaIII* restriction enzyme [ 11 ], respectively. The PCR program was similar to ITS1-5.8 SrDNA- ITS2 PCR with an annealing temperature of 57 °C.

### Data analysis

The statistical analysis was performed in SPSS software (version 22.0) for Windows (SPSS Inc., Chicago, IL, USA). Descriptive statistics are given by means and 95% confidence intervals for normally distributed data. It should be mentioned that categorical data were subsumed by relative frequencies. In analytical statistics, variables were compared between groups using the t-test. The results of the tests were considered significant for p-values of less than 0.05.

## Results

In total, 32 (53.33%) out of the 60 urine samples were culture positive for *Candida* spp. The age range of the participants was between ≤ 1 month to 12 years. There was no significant difference between the cases in terms of age and gender (male: 53.5% vs. female: 46.5%) (*P*>0.05). There was evidence of empiric antifungal treatment based on medical records, including itraconazole (5%), fluconazole (20%), amphotericin B (13%), and fluconazole was not qualified for antifungal therapy. Moreover, 19 (45.23%) subjects had hematological malignancy, 2 (7.1%) of them had heart disease, and the rest of them had other non-leukemia-related diseases. The evidence of neutropenia (45%), low weight (<1500) (21%), chemotherapy (7%), history of surgery (10%), antibiotic therapy use (17%) were also observed. The mean hospital stay was 21 days; more than 90% of the patients had urinary catheters, and about 26% of them received antibiotics. Overall, *C. albicans* (28.125%) and non-*C. albicans* spp. (71.87%) were identified using conventional chromogenic media. All the primarily identified *Candida* on CHROM agar were precisely differentiated using the PCR method.

**Figure 1:**
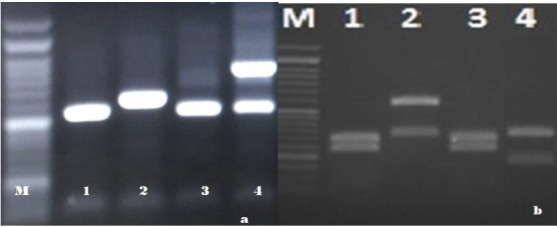
Agaros gel electrophoresis pattern of polymerase chain reaction products of *Candida*. a: before digestion with the restriction enzyme, lane 1-8: bands are all located between 524 and 871 bp which suggests that they possibly belonged to the *Candida* genus. b: enzymatic digestion pattern with *MspI* restriction enzyme for differentiation of *Candida* spp. a: *C. albicans* (297-238 bp): 1, 3; *C. glabrata* (314-557 bp): 2; *C. tropicalis* (184-340 bp):4; M: 100 bp DNA size marker.

**Figure 2:**
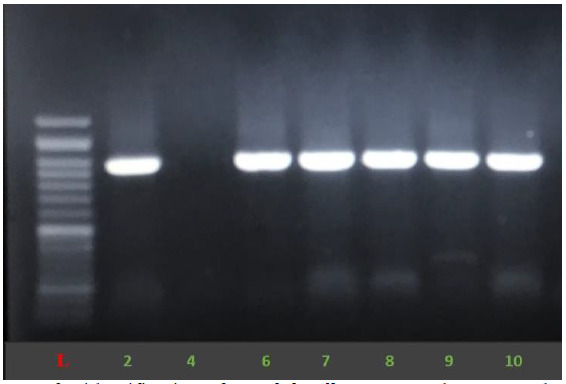
Amplification pattern of *HWP1* genes for identification of *Candida albicans* complex. L: 100 bp DNA size marker. The lanes in the picture belong to *Candida albicans* (900 bp).

**Figure 3:**
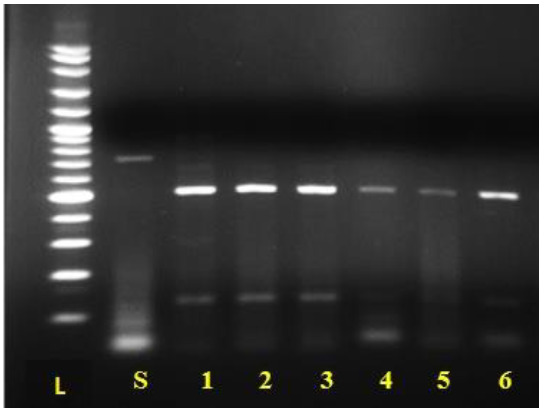
Identification of *Candida* parapsilosis complex after digestion with *NlaIII* restriction enzyme. L: 100 bp DNA size marker. Lane 1. *C. parapsilosis* complex before digestion; lanes 1-6. *C. parapsilosis* spp.

The bands were all located between 524 and 871 bp which suggested that they possibly belonged to *Candida* genus (Figure 1a). The RFLP fingerprint analysis revealed that *C. parapsilosis* (n=17; 53.125 %) was the most prevalent isolated species followed by *C. albicans* (n=9; 28.125%), C. tropicalis (n=4; 12.5%), and *C. glabrata* (n=2; 6.25%) (Figure1b). All of *C. albicans* complex strains were identified as *C. albicans*, based on amplification of *HWP1* gene (Figure 2). Further analysis of *C. parapsilosis* complex strains showed that all identified species were known as *C. parapsilosis* (Figure 3).

## Discussion

In this study, there was no statistically significant difference between the positive cases and age (*P*>0.05). This finding is in line with those of a study performed by Zarei et al. [ 12 ]. In another study, the UTI of the subjects represented the most frequent diagnosis (62%) followed by fungaemia (34%) and peritonitis (4%). In the aforementioned study, UTIs were more frequent in patients with urinary tract malformation, and most of the infections were reported among neonates [ 13 ].

In this study, 53.5% and 46.5% of the patients diagnosed with candiduria were male and female, respectively. In another study, Zarei et al. reported the frequency of in males and females to be 59.2% and 40.8%, respectively [ 12 ]. Similarly, Jain et al. and Gholamipour reported that candiduria was more common in males (68%) than females (32%) [ 14 , 15 ]. In another study, UTI was evaluated in 301 infants (1.1% of admissions, 253 males and 48 females with a ratio of 5.3:1), and only one infant had candiduria [ 16 ].

Regarding underlying diseases in this study, 45.23% of the patients had a hematological malignancy, 7.1% (n=2) of them had heart disease, and the rest of them had other non-leukemia-related diseases. According to Robinson et al., the most important predisposing factors in creating candiduria were hospitalization in intensive care units (76.2%) and antibiotic therapy [ 17 ]. Robinson et al. reported that the mortality rate due to candiduria in neonates in the neonatal intensive care unit was significant (30%) [ 17 ].

In this study, evidence of neutropenia, low weight (<1500 g), long hospitalization, chemotherapy, history of surgery, and antibiotic therapy could be considered risk factors. Regarding the risk factors, there was no significant difference between the two groups of candidiasis in terms of *C. albicans* and non-*albicans* *Candida* spp. (NAC) (*P*<0.01). In a study carried out by Gholamipour et al., long-term indwelling urinary drainage tools existed in 40% of the neonates with candiduria [ 14 ]. Platt et al. showed that 26.5% of all fungal urinary infections were associated with the application of indwelling catheters [ 18 ].

Regarding higher resistance to fluconazole in NAC spp., identification of *Candida* spp. is necessary for appropriate management of infection. Contrary to this study, in the study performed by Zarei et al., *C. albicans* (65.5%) was the most isolated species, followed by *C. glabrata* [ 19 ]. Moreover, in another research, *C. albicans* was the most common pathogen in neonate patients with candiduria followed by *C. parapsilosis* [ 20 ]. In another study, *C. albicans* was responsible for 97% of positive cultures in children [ 21 ]. Previously, we have reported the frequency of candiduria in 16.5% of hospitalized patients in two general hospitals in Ahvaz whose most common agent was *C. albicans* [ 19 ]. 

## Conclusion

In this study, *C. parapsilosis* and *C. albicans* were the most commonly observed species. *Candida* spp., which were once considered harmless, can now cause candidemia and significant morbidity and mortality in patients residing in intensive care units. Therefore, the identification of their underlying conditions and risk factors must be taken seriously. Furthermore, cases with more than one species of *Candida* and also the cases with bacterial infection require more attention, investigation, and consideration. 

## Acknowledgments

The authors of this article would like to appreciate all the subjects who participated in this project. 

## Authors’ contribution

Study concept, design, and technical supervision: A.F.; collection the samples from patients and interpretation: F.SH.; acquisition of data and drafting of the manuscript: A.F. and F.SH.; critical revision of the manuscript and scientific consultation: A.F.

## Financial disclosure

This research was financially supported by Shahid Beheshti University of Medical Sciences, Tehran, Iran.

## Conflicts of interest 

The authors declare that there were no conflicts of interest.
